# B cell and aquaporin‐4 antibody relationships with neuromyelitis optica spectrum disorder activity

**DOI:** 10.1002/acn3.52171

**Published:** 2024-09-02

**Authors:** Jeffrey L. Bennett, Sean J. Pittock, Friedemann Paul, Ho Jin Kim, Sarosh R. Irani, Kevin C. O'Connor, Kristina R. Patterson, Michael A. Smith, Michele Gunsior, Nanette Mittereder, William A. Rees, Daniel Cimbora, Bruce A. C. Cree

**Affiliations:** ^1^ Departments of Neurology and Ophthalmology Programs in Neuroscience and Immunology, University of Colorado School of Medicine, Anschutz Medical Campus Aurora Colorado USA; ^2^ Neurology, Laboratory Medicine and Pathology Center for Multiple Sclerosis and Autoimmune Neurology, Mayo Clinic Rochester Minnesota USA; ^3^ Experimental and Clinical Research Center Max Delbrueck Center for Molecular Medicine and Charité – Universitätsmedizin Berlin Germany; ^4^ Department of Neurology Research Institute and Hospital of National Cancer Center Goyang Republic of Korea; ^5^ Oxford Autoimmune Neurology Group, Nuffield Department of Clinical Neurosciences University of Oxford Oxford UK; ^6^ Department of Neurology Mayo Clinic Jacksonville Florida USA; ^7^ Department of Neurology and Immunobiology Yale School of Medicine New Haven Connecticut USA; ^8^ Horizon Therapeutics (now Amgen Inc., Thousand Oaks, California, USA) Gaithersburg Maryland USA; ^9^ UCSF Weill Institute for Neurosciences, Department of Neurology University of California San Francisco San Francisco California USA

## Abstract

This post hoc analysis of the randomized, placebo‐controlled N‐MOmentum study (NCT02200770) of inebilizumab in neuromyelitis optica spectrum disorder (NMOSD) evaluated relationships between circulating B‐cell subsets and aquaporin‐4 immunoglobulin G (AQP4‐lgG) titers and attacks. Among participants receiving placebo, CD20^+^ and CD27^+^ B‐cell counts were modestly increased from the pre‐attack visit to attack; plasmablast/plasma cell gene signature was increased from baseline to the pre‐attack visit (*p* = 0.016) and from baseline to attack (*p* = 0.009). With inebilizumab treatment, B‐cell subset counts decreased and did not increase with attacks. No difference in change of AQP4‐IgG titers from baseline to time of attack was observed.

## Introduction

B cells figure prominently in the pathophysiology of neuromyelitis optica spectrum disorder (NMOSD).^1^ All circulating B cells, plasmablasts (PBs), and some plasma cells (PCs) express surface CD19.^1^ CD19^+^ B cells produce pathogenic aquaporin‐4 immunoglobulin G (AQP4‐IgG) that can induce complement‐dependent cytotoxicity^2^, ^3^; however, prior studies found no correlation between either AQP4‐IgG titers or complement activity and NMOSD attacks/severity with the exception of one retrospective study that showed a possible correlation of clinical disease activity and AQP4 antibody serum levels.^4^, ^5^, ^6^ In vitro, circulating CD19^+^ B cells produced by germinal center activity secrete AQP4‐IgG^7^, ^8^ which is linked to NMOSD disease activity. Hence, inebilizumab, a humanized, affinity‐optimized, anti‐CD19 monoclonal antibody approved to treat NMOSD in AQP4‐IgG–seropositive adults, is well‐placed to mediate paralleled serological benefits.^1^, ^9^ The randomized, placebo‐controlled, phase 2/3 N‐MOmentum study showed risk of NMOSD attack was significantly lower with inebilizumab versus placebo (hazard ratio, 0.272; *p* < 0.0001),^10^ with efficacy maintained for over 4 years (annualized attack rate, 0.052 attacks/person‐years).^11^ The current study correlates B‐cell subsets (including PBs/PCs and CD27^+^ memory B cells) and AQP4‐IgG titers with disease activity in the N‐MOmentum trial including an evaluation of therapeutic impact on biomarkers of disease activity.

## Methods

Study details are included in Supplementary Methods or were previously reported.^10^, ^12^ Briefly, in the N‐MOmentum study (NCT02200770), patients with NMOSD were randomized 3:1 to inebilizumab or placebo.^10^, ^12^ The study protocol was reviewed by an institutional review board, and all participants provided informed consent.^10^ B‐cell subsets were analyzed at baseline, at time of attack, and the study visit preceding attack by flow cytometry using fresh whole blood. PB/PC gene signature was assessed by qRT‐PCR analysis of blood RNA samples.^13^, ^14^ AQP4‐IgG titers were measured in serum using a live cell‐based flow cytometry assay as previously described^4^ and KRONUS immunoassay.

## Results

### Participants

Among the 231 participants enrolled in N‐MOmentum, 21/174 (12%) who received inebilizumab and 22/56 (39%) who received placebo experienced NMOSD attacks during the randomized controlled period (RCP).^10^ During the open‐label period, 31/216 participants (14.4%) experienced attacks. The median (IQR) time between attack and the preceding visit was 22 (13–29) days in the placebo cohort and 28 (18–50) days in the inebilizumab cohort.

### CD20 ^+^ B cells, CD27 ^+^ memory B cells, and PB/PC signature

**Figure 1:**
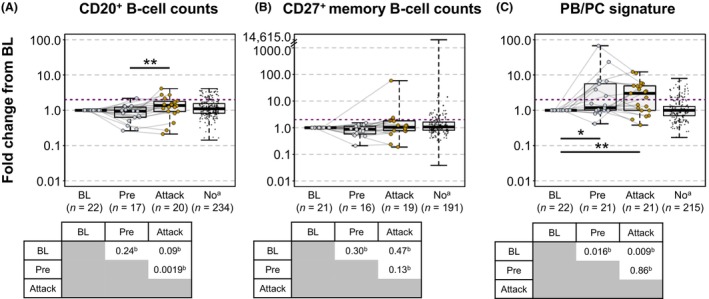
CD20^+^ B‐cell counts, CD27^+^ memory B‐cell counts, and PB/PC signature among participants receiving placebo during the N‐MOmentum study. BL, baseline; PB, plasmablast; PC, plasma cell. Results expressed as fold change from BL for (A) CD20^+^ B cells, (B) CD27^+^ memory B cells, and (C) PB/PC signature. Data presented at BL, before attack (pre), and during attack, or for participants not experiencing an attack (no). Lines between points indicate individual patient profiles. Dashed red line indicates a 2‐fold change from baseline. The n values correspond to individual samples; each patient may have >1 sample and/or may be included in multiple groups. ^a^Samples from participants with no attacks are included for reference. ^b^Paired fold change from baseline cell counts or PB/PC signature measurements at each timepoint (BL, pre, attack) were analyzed using a Wilcoxon signed rank test. *p < 0.05. **p < 0.01.

In participants who experienced an attack while receiving placebo, overall CD20^+^ B‐cell counts modestly increased from the visit preceding attack (median [IQR] fold change from baseline, 0.9 [0.6–1.1]) to the time of attack (1.4 [0.9–1.8]; *p* = 0.002 based on pairwise comparisons of absolute counts; Fig. 1A; Table S1), whereas CD27^+^ memory B‐cell counts were not significantly increased from the visit preceding attack (median [IQR] fold change from baseline, 0.9 [0.6–1.0]) to the time of attack (1.0 [0.8–1.8]; *p* = 0.13 based on pairwise comparisons of absolute counts; Fig. 1B; Table S1). CD20^+^ B cells increased ≥2‐fold from baseline in 20% (4/20) of attack samples and 16% (37/234) of non‐attack samples, and CD27^+^ memory B cells increased >2‐fold from baseline in 16% (3/19) of attack samples and 19% (36/191) of non‐attack samples. In contrast, naïve B cells (CD20^+^CD27‐IgD^+^) in placebo participants increased significantly from baseline to the time of attack (*P* = 0.01) and from the visit preceding attack to the time of attack (*p* = 0.001; Fig. S1). PB/PC signature was already increased from baseline at the visit preceding attack (*p* = 0.016), and a significant increase in PB/PC signature was seen at the time of attack compared with baseline (*p* = 0.009). However, PB/PC signature during an attack was not significantly different from the preceding visit (Fig. 1C; Table S1). PB/PC signature increased >2‐fold from baseline in 57% (12/21) of attack samples vs 16% (35/215) of non‐attack samples (*p* = 0.02).

Compared with placebo, inebilizumab provided rapid PB/PC depletion that was sustained through the RCP based on both flow cytometry and gene expression signature (Fig. S2) as previously reported.^13^ Among participants treated with inebilizumab, CD20^+^ B‐cell counts, CD27^+^ memory B‐cell counts, naïve B cells, and PB/PC signature decreased significantly from baseline at the time of attack and pre‐attack visits, with no significant differences between the two visits; no samples from participants who experienced attacks showed increases from baseline or pre‐attack samples (Fig. S3; Table S1).

### AQP4‐IgG titers

**Figure 2:**
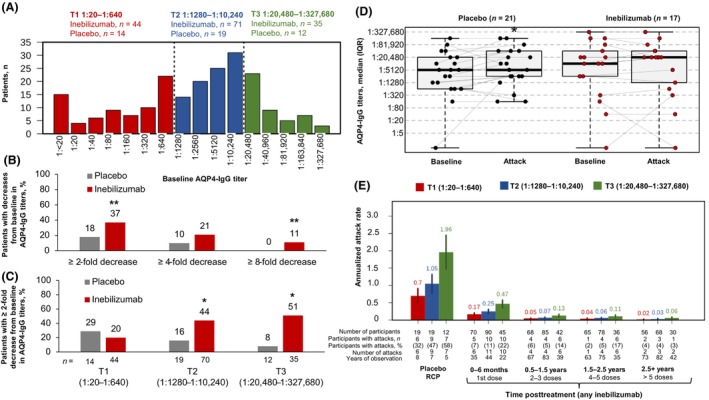
AQP4‐IgG titers during the N‐MOmentum study. AQP4‐IgG, aquaporin‐4 immunoglobulin G; IQR, interquartile range; RCP, randomized controlled period; SEM, standard error of the mean; T, tertile. (A) Histogram of baseline AQP4‐IgG titers by tertile among participants with baseline titers >1:20. (B) The percentage of participants experiencing a ≥2‐fold, ≥4‐fold, and ≥8‐fold decrease in AQP4‐IgG titers at the end of the RCP in the placebo and inebilizumab groups. *P* values generated from Fisher's exact test for the proportion of participants with decreases in titers surpassing each cutoff. (C) The percentage of participants with baseline titers >1:20 who experienced a ≥2‐fold decrease in AQP4‐IgG titers at the end of the RCP stratified by baseline AQP4‐IgG tertile in the placebo and inebilizumab groups. *p* values generated from Fisher's exact test for the proportion of participants with decreases in titers surpassing each cutoff. (D) Box plots of AQP4‐IgG titers for participants receiving placebo or inebilizumab at baseline and time of attack. *p* values for within‐group differences vs baseline were calculated using Wilcoxon signed rank test, while *p* values for differences between the placebo vs inebilizumab groups were calculated using Mann–Whitney *U* test. (E) Annualized attack rate over time by AQP4‐IgG tertiles at Day 1 of the RCP. Data presented as mean (SEM) estimated by Poisson regression. *p < 0.05. **p < 0.01.

Among AQP4‐IgG–seropositive participants, baseline AQP4‐IgG titer tertiles were 1:20–1:640 (T1), 1:1280–1:10,240 (T2), and 1:20,480–1:327,680 (T3; Fig. 2A). At the end of the RCP, 59/159 (37%) of inebilizumab‐treated participants vs 9/50 (18%) of placebo‐treated participants had a ≥2‐fold decrease in AQP4‐IgG titers (*p* = 0.014, Fisher's exact test), and 11% vs 0% had a ≥8‐fold decrease (*p* = 0.008, Fisher's exact test; Fig. 2B). Among participants with baseline AQP4‐IgG titers >1:20,480, AQP4‐IgG titers decreased ≥2‐fold from baseline in 51% (18/35) of those treated with inebilizumab vs 8% (1/12) of those who received placebo (*p* < 0.05; Fig. 2C). The ELISA‐based KRONUS assay correlated well with the flow cytometry assay (Pearson r = 0.78, Fig. S4); no significant decreases in the KRONUS AQP4 measurements were observed with continued rounds of inebilizumab treatment in the open‐label period.

AQP4‐IgG titers increased from baseline during attack in 11/21 (52%) participants receiving placebo (*p* = 0.024; Fig. 2D); however, AQP4‐IgG titers increased during attack in 6/17 (35%) participants receiving inebilizumab and this difference was not significantly different from baseline (*p* = 0.76). Even though an increase in AQP4‐IgG titers was observed in the placebo but not the inebilizumab group at time of attack, changes in AQP4‐IgG titers from baseline to the time of attack were not significantly different between the placebo and inebilizumab treatment groups (*p* = 0.15).

Within the placebo group, 85% of participants had a ≥2‐fold increase from baseline to the time of attack in PB/PC gene signature and/or AQP4‐IgG titers; 25% of participants had a ≥2‐fold increase from baseline in both (Fig. S5). Among samples (*n* = 215) drawn from participants who received placebo and did not experience an attack during the RCP, 32% of samples had a 2‐fold increase from baseline in PB/PC signature and/or AQP4‐IgG titers, and 67.9% had neither.

Increased annualized attack rate was observed in placebo‐ and inebilizumab‐treated participants within the highest tertile of AQP4‐IgG titers at Day 1 of the RCP (≥1:20,480; *p* = 0.0004), and progressive decreases in annualized attack rate were observed with continued rounds of inebilizumab treatment across all subgroups during the open‐label period (Fig. 2E).

**Figure 3:**
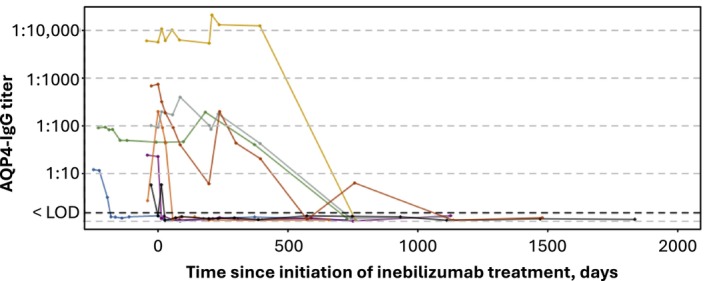
Longitudinal analysis of AQP4‐IgG titers. AQP4‐IgG, aquaporin 4 immunoglobin G; LOD, level of detection. Titers were measured over time among participants who were AQP4‐IgG seropositive at baseline and had undetectable levels of AQP4‐IgG while receiving inebilizumab during the N‐MOmentum study. One participant (in orange) experienced an attack at D85 post treatment. This participant converted to seronegative via the titer assay by Day 57.

All participants who were AQP4‐IgG seronegative at baseline received inebilizumab and none developed AQP4‐IgG titers during the study. Eight participants (3.8%; 2 placebo/inebilizumab and 6 inebilizumab/inebilizumab) who were AQP4‐IgG seropositive at baseline (*n* = 213) had undetectable levels of AQP4‐IgG during the study (Fig. 3).

## Discussion

To our knowledge, the flow cytometric data presented herein is the largest analysis of B‐cell subsets in NMOSD to date. Surprisingly, a correlation between memory B cells and disease activity was not found. Rather, PBs and PCs appear to correlate with NMOSD disease activity. Furthermore, no difference in change of AQP4‐IgG titers from baseline to time of attack was observed in patients receiving either placebo or inebilizumab which supports evidence regarding limited utility of AQP4‐IgG titers for monitoring NMOSD disease activity.^4^, ^15^

Inebilizumab treatment substantially reduced CD20^+^ B cells, CD27^+^ memory B cells, naïve B cells, and PBs/PCs.^10^, ^13^ Among placebo‐treated participants, CD20^+^ B cells, PB/PC signature, and AQP4‐IgG titers increased from baseline during attacks. The PB/PC signature was also increased at the pre‐attack visit. While there was a trend toward increased CD27^+^ memory B‐cell counts from the visit preceding attack to the time of attack, there was no meaningful association between total CD20^+^ B cells or CD27^+^ memory B‐cell counts and attacks. While a previous study reported an association between elevated memory B cells and NMOSD relapse,^16^ in this study memory B cells were defined as CD19^+^CD27^+^, which could include PBs/PCs. In our study, memory B cells were defined as CD45^+^ (CD3^−^, CD14^−^, CD56^−^), CD20^bright^, CD19^+/−^, and CD27^+^ possibly accounting for the divergent findings between these studies.

Among participants who received placebo, the PB/PC signature was elevated at the time of attack relative to baseline. These results support previous findings suggesting that CD19^+^ PBs/PCs may contribute to NMOSD relapse.^2^, ^16^ Circulating B cells are likely generated by germinal center activity, supporting a link between B‐cell activation and clinical disease activity.^7^ Indeed, direct lymph node aspiration identified AQP4‐IgG and AQP4‐targeted B cells sensitive to B‐cell depletion therapy.^8^ CD19 is expressed on PBs and some PCs that do not express CD20.^1^, ^13^ Anti‐CD20 treatments (e.g., rituximab) do not deplete all CD19^+^ PBs^17^ or consistently affect serum AQP4‐IgG levels in patients with NMOSD,^8^ whereas, in this study, inebilizumab decreased AQP4‐IgG levels in a subset of participants, particularly those with high AQP4‐IgG titers at baseline. A clear correlation between higher AQP4 IgG titers and increased attack rates was observed in this study. Nonetheless, attack rate decreased over time regardless of AQP4 titer (Fig. 2E). Studies in muscle‐specific tyrosine kinase myasthenia gravis and IgG4‐related disease demonstrated some pathogenic PBs survive rituximab therapy and may be associated with relapse.^18^, ^19^, ^20^ In addition to targeting CD19^+^ PBs in the periphery, inebilizumab may be more effective in targeting PBs in bone marrow. Moreover, inebilizumab effectively depletes CD20^+^ B cells and PB/PC signature independent of FCGR3A genotype.^21^ Our findings further support targeting CD19^+^ B cells as a mechanism for potentially reducing the likelihood of attack or attack severity.

While the cause of NMOSD attacks is unknown, a correlation between PBs/PCs as well as AQP4‐IgG titers was observed with placebo but not with active treatment. This mirrors findings for other biomarkers in the N‐MOmentum trial (GFAP, neurofilament, etc.).^22^, ^23^ These results suggest that treatment status may impact the sensitivity of potential biomarkers of disease activity. Indeed, the blood compartment is only a surrogate for lymphoid tissue.

Current evidence supports a multifactorial etiology of NMOSD attacks, requiring blood–brain barrier permeability, AQP4‐IgG, both complement‐dependent and complement‐independent means of antibody‐mediated toxicity, pro‐inflammatory cytokines, etc. Moreover, we do not have insight into the B‐cell levels or AQP4‐IgG titers in the tissue, and recent evidence suggests that not all AQP4‐IgG are equally pathogenic.^7^, ^24^ In this study, B cell/B‐cell subset and PC gene signature were weakly correlated with AQP4 titers. Presumably, if large increases in PC signature were observed, increases in AQP4‐IgG would occur simultaneously or proceeding the increase in the PC signature. However, an evaluation of the eight participants who displayed the largest increases in the PC signature during the study showed that the increases in PC signature were largely not followed by increases in AQP4 IgG, (Fig. S7). While inebilizumab targets CD19^+^ PBs and some PCs, there are long‐lived CD19^−^ PCs that reside in tissue (e.g., bone marrow). It is possible that a significant proportion of AQP4‐IgG is produced by these CD19^−^ PCs. Interestingly, a small number of placebo patients (*n* = 9) showed a decrease in AQP4‐IgG titers (several with a low baseline AQP4‐IgG) which may be attributed to natural fluctuation within tissue resident PC niches. Further studies will be needed to better understand associations between B‐cell subsets and AQP4‐IgG titers within lymphoid tissues. Other important limitations include the post hoc approach that is hypothesis generating, the low total number of attacks, the short duration of placebo exposure, and the absence of an independent confirmatory dataset.

In conclusion, B‐cell subsets (particularly those with a PB/PC gene signature) and AQP4‐IgG levels were increased in a significant proportion of untreated participants at the time of attack. Inebilizumab treatment reduced B‐cell subset numbers, including PB/PC and AQP4‐IgG titers, impacting relapse risk, which supports targeting CD19^+^ B cells as a mechanism for reducing the likelihood of attacks.

## Funding Information

Provided by Horizon Therapeutics PLC (now Amgen Inc.).

## Author Contributions

**JLB**, **SJP**, **SRI**, **KCO**, **MAS**, **MG**, **NM**, **WAR**, **KRP**, **DC,** and **BACC** contributed to the study concept and design. **SJP** and **BACC** contributed to data acquisition. **NM** performed gene signature analysis. **MAS** performed statistical analysis. All authors contributed to data interpretation and provided their critical review and final approval of the manuscript.

## Conflict of Interest

**JLB** has received study design/consultation fees from MedImmune/Viela Bio (now Amgen) and personal fees from AbbVie, Alexion, Antigenomycs, BeiGene, Chugai, Clene Nanomedicine, EMD Serono, Genentech, Genzyme, Mitsubishi Tanabe Pharma, Novartis, Reistone Bio, Roche, Imcyse, and TG Therapeutics. He has received grants from the National Institutes of Health, Novartis, and Mallinckrodt, and holds a patent for Aquaporumab. **SJP** has received grants, personal fees, and nonfinancial support from Alexion; grants from Autoimmune Encephalitis Alliance; grants, personal fees, nonfinancial support, and other support from MedImmune and Viela Bio (now Amgen); grants, personal fees, nonfinancial support, and other support from Roche, Genentech; consulting support from Astellas; and personal fees for consulting services from UCB. He holds the following patents that are relevant to this work: Patent #8889102 (Application #12‐678350, Neuromyelitis Optica Autoantibodies as a Marker for Neoplasia) and Patent #9891219B2 (Application #12‐573942, Methods for Treating Neuromyelitis Optica [NMO] by Administration of Eculizumab to an Individual that is Aquaporin‐4[AQP4]‐IgG Autoantibody Positive) for which he has received royalties. He works as a consultant in the Mayo Clinic Neuroimmunology Laboratory clinical service. The Mayo Clinic Neuroimmunology Laboratory commercially offers AQP4‐IgG testing, but revenue accrued does not contribute to salary, research support, or personal income. **FP** has received research support, speaker fees, and travel reimbursement from Bayer, Biogen Idec, Merck Serono, Novartis, Sanofi Genzyme, and Teva. He is supported by the German Competence Network for Multiple Sclerosis and the German Research Council (DFG Exc 257). He has received travel reimbursement from the Guthy–Jackson Charitable Foundation and serves on the steering committee of the OCTIMS study sponsored by Novartis. **HJK** received a grant from the National Research Foundation of Korea and research support from Aprilbio, Eisai, and UCB, and has received consultancy/speaker fees from Alexion, Altos Biologics, AstraZeneca, Biogen, Daewoong Pharmaceutical, Eisai, GC Pharma, Handok Pharmaceutical, Kaigene, Kolon Life Science, MDimune, Merck Serono, Mitsubishi Tanabe Pharma, Roche, and Sanofi Genzyme. He is a coeditor for the *Multiple Sclerosis Journal* and an associated editor for the *Journal of Clinical Neurology*. **SRI** has received honoraria and/or research support from ADC Therapeutics, Cerebral Therapeutics, CSL Behring, Immunovant, Janssen, MedImmune, ONO Pharma, Roche, and UCB. He receives royalties on a licensed patent application for LGI1/CASPR2 testing. This research was funded in whole or in part by a senior clinical fellowship from the Medical Research Council [MR/V007173/1], Wellcome Trust fellowship [104079/z/14/z] and by the National Institute for Health Research (NIHR) Oxford Biomedical Research Centre (BRC). For the purpose of open access, the author has applied a cc by public copyright license to any author accepted manuscript (AAM) version arising from this submission. The views expressed are those of the author(s) and not necessarily those of the NHS, the NIHR or the Department of Health. **KCO** has received research support from Alexion (now AstraZeneca), argenx, Cabaletta Bio, Ra Pharma (now UCB), and Viela Bio (now Amgen). He is a consultant and equity shareholder of Cabaletta Bio. He has received compensation for serving as a consultant/advisor/speaker for Alexion (now AstraZeneca), IgM Biosciences, Roche/Genentech, and UCB. **KRP, MAS**, **MG**, **NM, WAR**, and **DC** are current (**KRP**, **MAS**, **NM**, **WAR**, **DC**) or former (**MG**) full‐time employees of Horizon Therapeutics (now Amgen) and may hold Horizon/Amgen stock and/or stock options. **BACC** has received personal compensation for consulting from Alexion, Atara, Autobahn, Avotres, Biogen, Boston Pharma, EMD Serono, Gossamer Bio, Hexal/Sandoz, Horizon (now Amgen), Immunic AG, Neuron23, Novartis, Sanofi, Siemens, and TG Therapeutics. He has received research support from Genentech.

## Supporting information


Appendix S1.

## Data Availability

There is a plan to share data. This may include de‐identified individual patient data for variables necessary to address the specific research question in an approved data‐sharing request; also related data dictionaries, study protocol, statistical analysis plan, informed consent form, and/or clinical study report. Data sharing requests relating to data in this manuscript will be considered after the publication date and (1) this product and indication (or other new use) have been granted marketing authorisation in both the USA and Europe, or (2) clinical development discontinues and the data will not be submitted to regulatory authorities. There is no end date for eligibility to submit a data sharing request for these data. Qualified researchers may submit a request containing the research objectives, the Amgen product(s) and Amgen study or studies in scope, endpoints or outcomes of interest, statistical analysis plan, data requirements, publication plan, and qualifications of the researchers. In general, Amgen does not grant external requests for individual patient data for the purpose of re‐evaluating safety and efficacy issues already addressed in the product labelling. A committee of internal advisors reviews requests. If not approved, requests might be further arbitrated by a Data Sharing Independent Review panel. Requests that pose a potential conflict of interest or an actual or potential competitive risk might be declined at Amgen’s sole discretion and without further arbitration. Upon approval, information necessary to address there search question will be provided under the terms of a data sharing agreement. This may include anonymised individual patient data or available supporting documents, or both, containing fragments of analysis code where provided in analysis specifications. Further details are available online. For more information, or to submit a request, please email medinfo@amgen.com
