# Supplementary material for: B cell and aquaporin‐4 antibody relationships with neuromyelitis optica spectrum disorder activity
Source: Ann Clin Transl Neurol. 2024 Sep 2;11(10):2792–8. doi: 10.1002/acn3.52171 (PMC11514900; doi:10.1002/acn3.52171)

**Supporting information**

**Supplementary methods**

## Participants

N-MOmentum was a phase 2/3, randomized, placebo-controlled study to evaluate the efficacy and safety of inebilizumab monotherapy for the treatment of neuromyelitis optica spectrum disorder (NMOSD; NCT02200770); the study design was previously reported in detail. ^1,2^ Patients diagnosed with NMOSD were randomized 3:1 to receive inebilizumab 300 mg or placebo intravenously on days 1 and 15. The study included a randomized controlled period of up to 28 weeks (or until an adjudicated NMOSD attack) followed by an optional open-label period of ≥ 2 years in which participants received inebilizumab 300 mg every 26 weeks. ^2,3^ All participants provided written informed consent. ^2^

## Assessments

Absolute B-cell counts were assessed by flow cytometry using fresh whole blood. Memory B cells were defined as CD45^+^ (CD3^−^, CD14^−^, CD56^−^), CD20^bright^, CD19^+/−^, and CD27^+^. Plasmablast (PB)/plasma cell (PC) gene signature was assessed using qRT-PCR analysis from RNA blood samples for 4 genes predominantly expressed in circulating PCs (*IGHA1*, *IGJ*, *IGKV4-1*, and *TNFRSF17*). ^4^

All aquaporin-4 immunoglobulin G (AQP4-lgG) measurements were performed using serum. AQP4-IgG titers were determined using a live cell-based flow cytometry assay (validated and certified by the College of American Pathology) using the recombinant AQP4 M1 isoform with a GFP-tagged C-terminus. ^5^ Such cell-based assays have been shown to have an optimized sensitivity and specificity. ^5^ Samples were screened at 1:20 dilution and, if positive, retested at doubling dilution steps. The farthest dilution yielding a positive result (IgG binding index ≥ 2.0) was recorded as the end point of positivity. AQP4-IgG titers were also measured by KRONUS immunoassay.

## Statistical Analysis

B-cell subsets were analyzed at baseline, the time of attack, and the study visit preceding the attack. Paired fold-change B-cell counts or PB/PC signature measurements were analyzed using Wilcoxon signed rank tests. The proportion of participants with decreases in AQP4-IgG titers surpassing each fold-change cutoff were analyzed using Fisher’s exact tests. Within-group differences vs baseline AQP4-IgG titers were analyzed using Wilcoxon signed rank tests, while differences in AQP4-IgG titers between groups were analyzed using Mann-Whitney U tests.

**Supplementary references**

1. Cree BA, Bennett JL, Sheehan M, et al. Placebo-controlled study in neuromyelitis optica-ethical and design considerations. Mult Scler. 2016;22(7):862-872.

2. Cree BAC, Bennett JL, Kim HJ, et al. Inebilizumab for the treatment of neuromyelitis optica spectrum disorder (N-MOmentum): a double-blind, randomised placebo-controlled phase 2/3 trial. Lancet. 2019;394(10206):1352-1363.

3. Bennett JL, Aktas O, Rees WA, et al. Association between B-cell depletion and attack risk in neuromyelitis optica spectrum disorder: an exploratory analysis from N-MOmentum, a double-blind, randomised, placebo-controlled, multicentre phase 2/3 trial. EBioMedicine. 2022;86:104321.

4. Streicher K, Morehouse CA, Groves CJ, et al. The plasma cell signature in autoimmune disease. Arthritis Rheumatol. 2014;66(1):173-184.

5. Fryer JP, Lennon VA, Pittock SJ, et al. AQP4 autoantibody assay performance in clinical laboratory service. Neurol Neuroimmunol Neuroinflamm. 2014;1(1):e11.

**Table S1.** Median (IQR) fold change from baseline in B-cell subsets

| **Table S1.** B-cell subsets | | | | |
| --- | --- | --- | --- | --- |
| **B-cell subset, median (IQR) fold change from baseline** | **Placebo** | | **Inebilizumab** | |
|  | Visit preceding attack | Attack visit | Visit preceding attack | Attack visit |
| CD20^+^ B cells | 0.9 (0.6–1.1) | 1.4 (0.9–1.8) | 0.0053 (0.0016–0.031) | 0.0040 (0.00053–0.043) |
| CD27^+^ memory B cells | 0.9 (0.6–1.0) | 1.0 (0.8–1.8) | 0.0077 (0.0016–0.019) | 0.0037 (0.00099–0.036) |
| PB/PC signature | 1.5 (1.0–6.4) | 3.0 (1.0–4.9) | 0.048 (0.034–0.082) | 0.041 (0.017–0.11) |
| IQR, interquartile range; PB, plasmablast; PC, plasma cell. | | | | |

**Table S2**. Summary of the number of N-MOmentum participants included for each B cell subset analysis in both the Randomized Controlled Period and Open Label Period of the clinical study


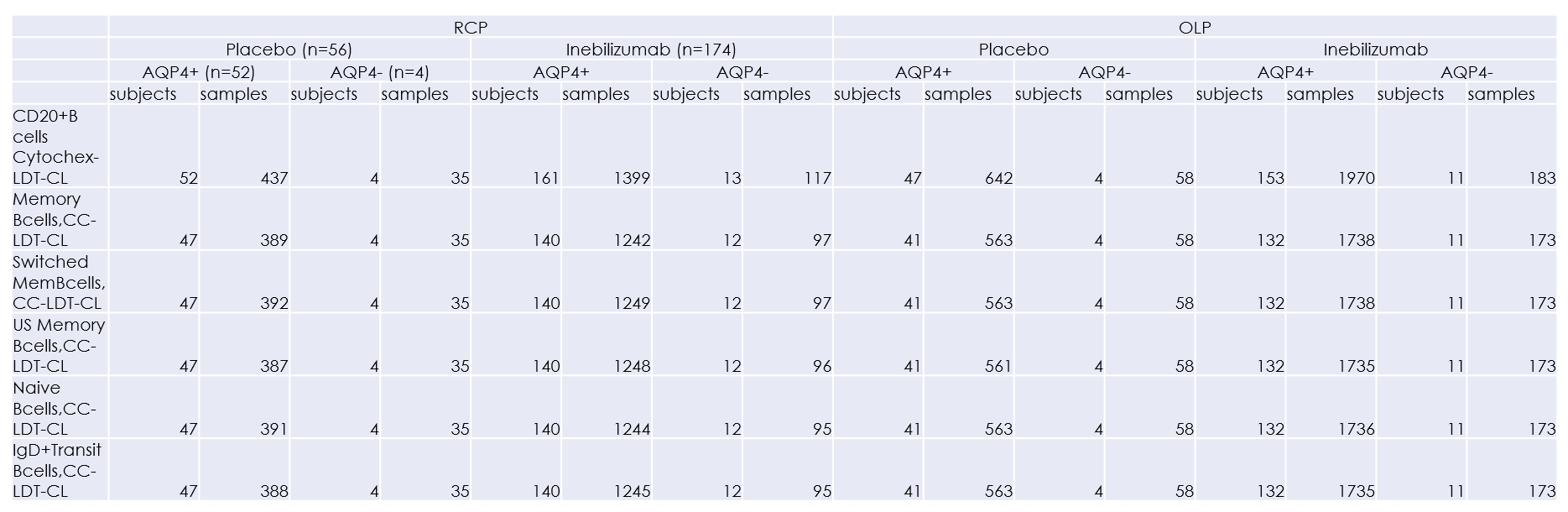


**Figure S1.** Naïve B-cell counts among participants receiving placebo or inebilizumab during the N-MOmentum study.

BL, baseline.

Naïve B-cell counts for participants receiving (A) placebo or (B) inebilizumab. Data presented at BL, before attack (pre), and during attack, or for participants not experiencing an attack (no). Results expressed as fold change from BL. Lines between points indicate individual patient profiles. The n values correspond to individual samples; each patient may have > 1 sample and/or may be included in multiple groups. ^a^Samples from participants with no attacks are included for reference. ^b^Paired absolute cell counts at each timepoint (BL, pre, attack) were analyzed using a Wilcoxon signed rank test.


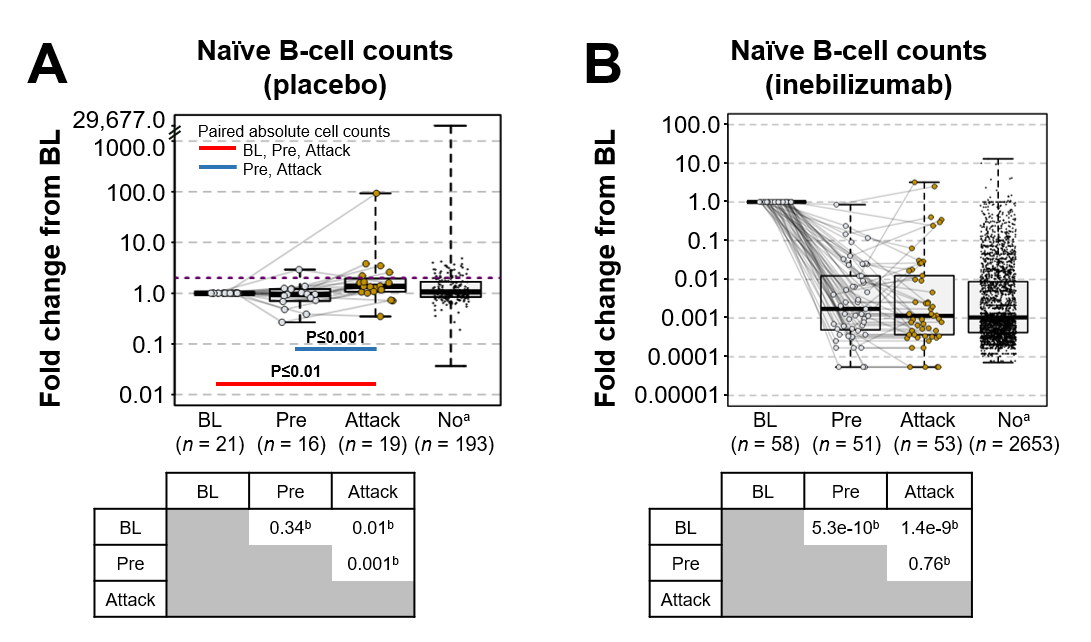


**Figure S2.** PB/PC counts during the randomized controlled period of the N-MOmentum study.

FACS, fluorescence-activated cell sorting; PB, plasmablast; PC, plasma cell; RCP, randomized controlled period.
(A) PB and PC counts assayed by FACS in participants receiving inebilizumab. (B) PB and PC counts assayed by gene expression signature in participants receiving placebo or inebilizumab. ****P* < 0.001.


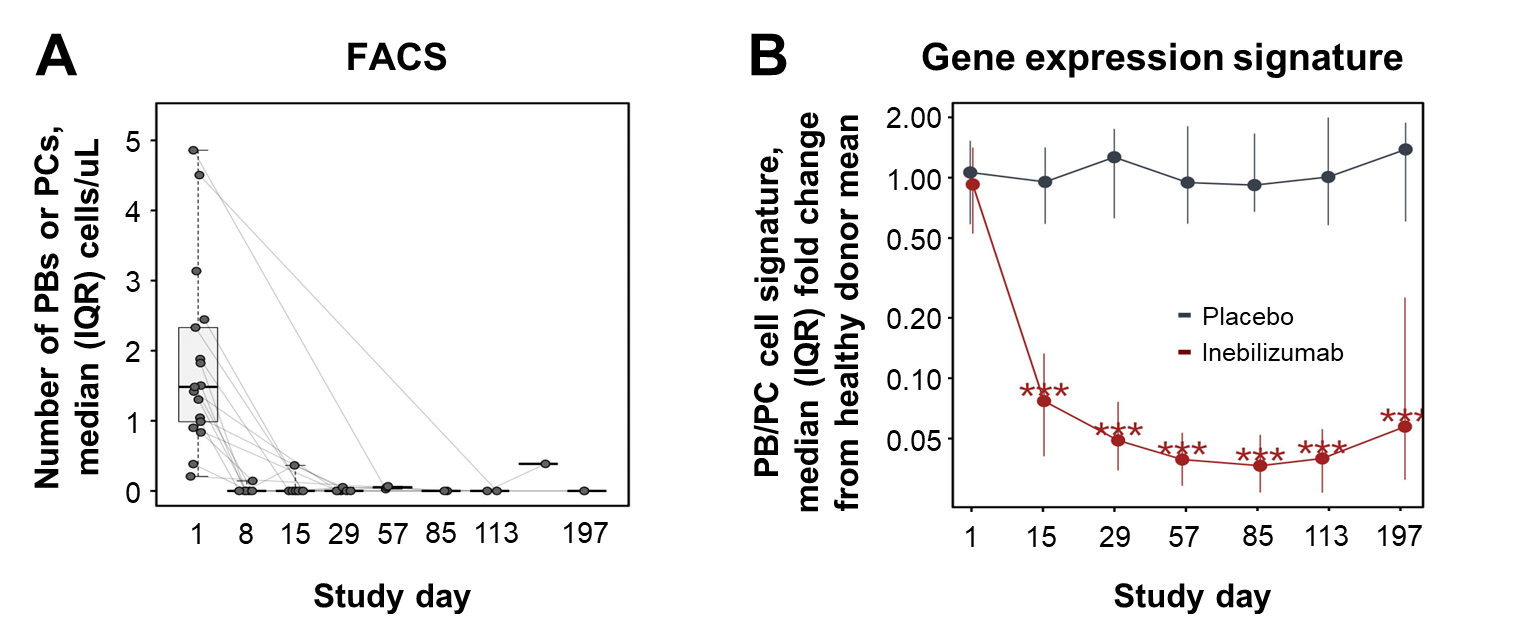


**Figure S3.** CD20^+^ B-cell counts, CD27^+^ memory B-cell counts, and PB/PC signature among participants receiving inebilizumab in the N-MOmentum study.

BL, baseline; PB, plasmablast; PC, plasma cell.
Results expressed as fold change from BL for (A) CD20^+^ B cells, (B) CD27^+^ memory B cells, and (C) PB/PC signature. Data presented at BL, before attack (pre), and during attack, or for participants not experiencing an attack (no). Lines between points indicate individual patient profiles. The n values correspond to individual samples; each patient may have more than 1 sample and/or may be included in multiple groups. ^a^Samples from participants with no attacks are included for reference. ^b^Paired absolute cell counts or PB/PC signature measurements at each timepoint (BL, pre, attack) were analyzed using a Wilcoxon signed rank test. ****P* < 0.001.


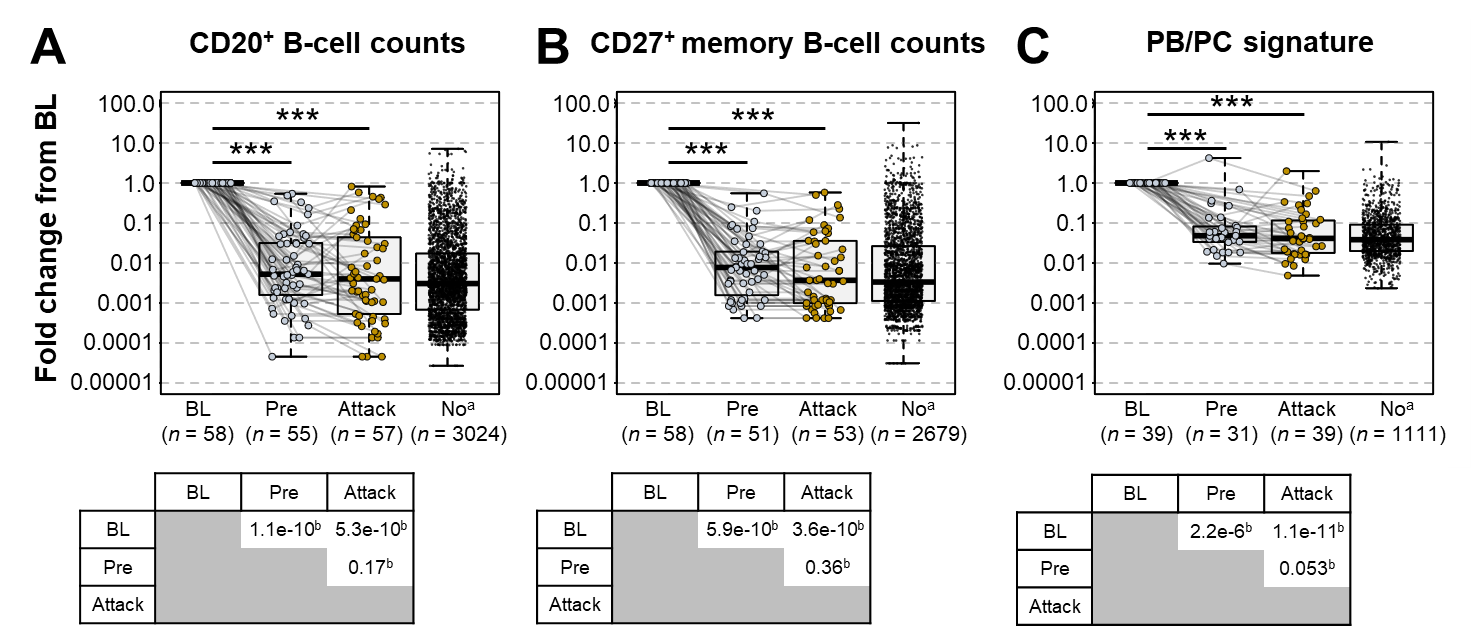


**Figure S4.** AQP4-IgG titers in the open-label period.

AQP4-IgG, aquaporin-4 immunoglobin G; IU, international units; OLP, open-label period T, tertile.
(A) Proportion of participants in the inebilizumab and placebo cohorts who displayed decreases and increases in AQP4-IgG titers at weeks 52 and 104 of the OLP. (B) Proportion of participants in any inebilizumab group who displayed decreases in AQP4-IgG titers across baseline AQP4-IgG tertiles at weeks 52 and 104 post-inebilizumab treatment. (C) (C) KRONUS AQP4-IgG quantitation in any inebilizumab group vs weeks posttreatment across AQP4-IgG tertiles (median ± IQR). (D) AQP4 titers averaged from the 2^nd^ dosing interval onward also correlate with NMOSD attack rates over 2.5+ years of inebilizumab treatment.


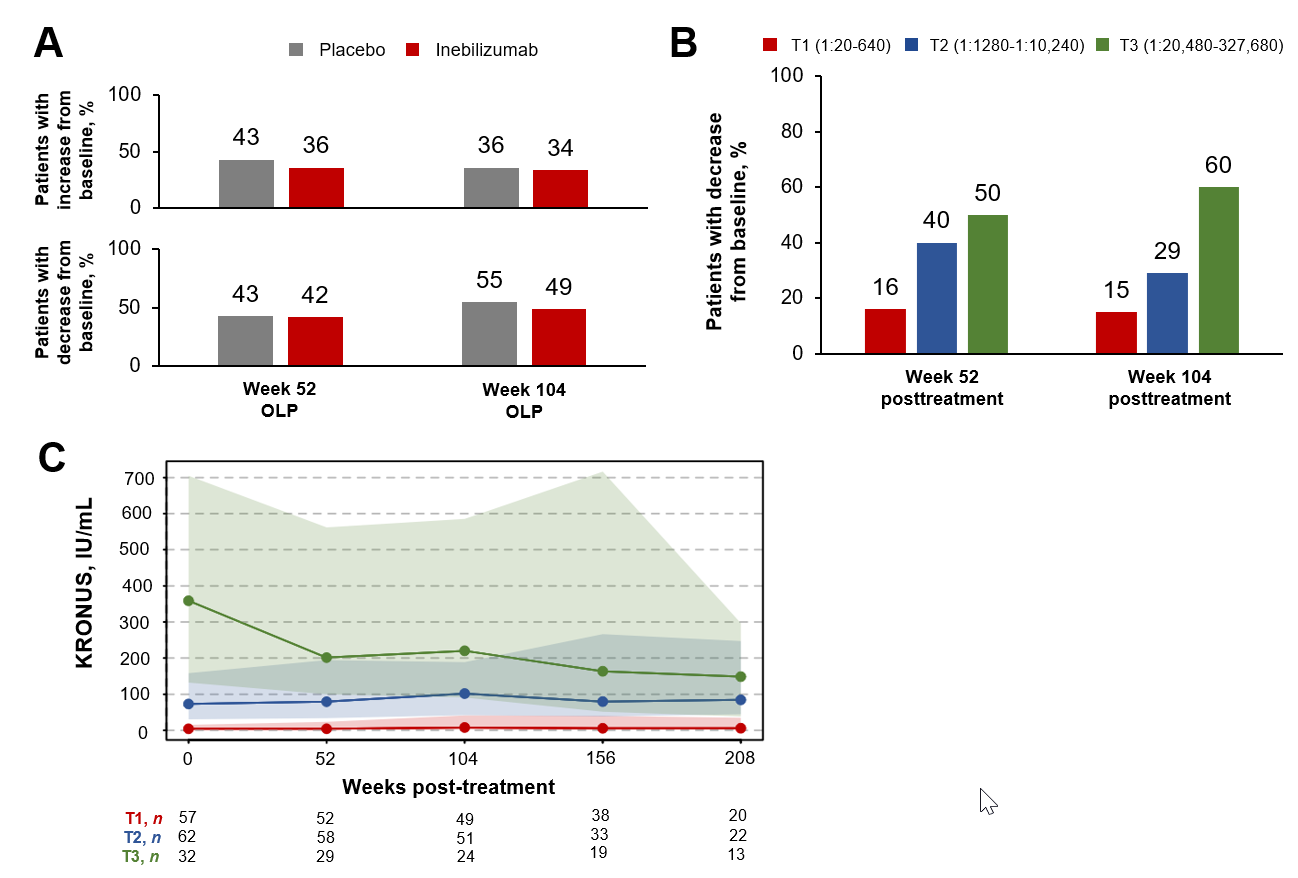


**D**
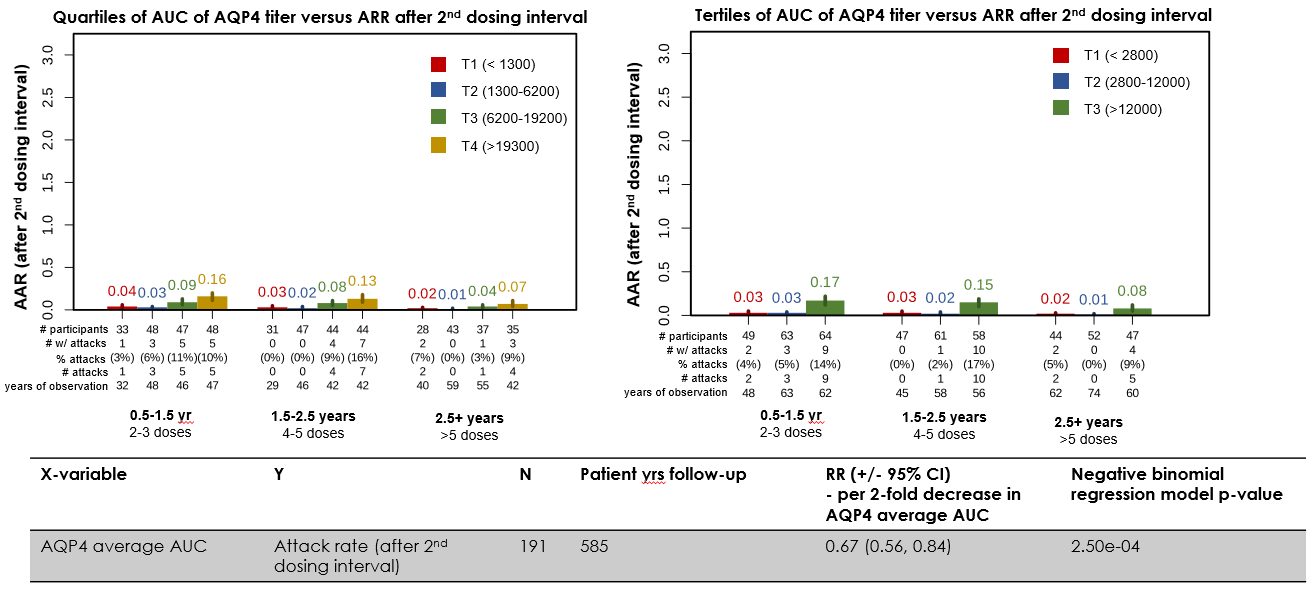


**Figure S5.** Correlation of fold change from baseline in AQP4-IgG titers and PB/PC signature at the time of attack in participants receiving placebo during the N-MOmentum study.

AQP4-IgG, aquaporin-4 immunoglobin G; PB, plasmablast; PC, plasma cell.


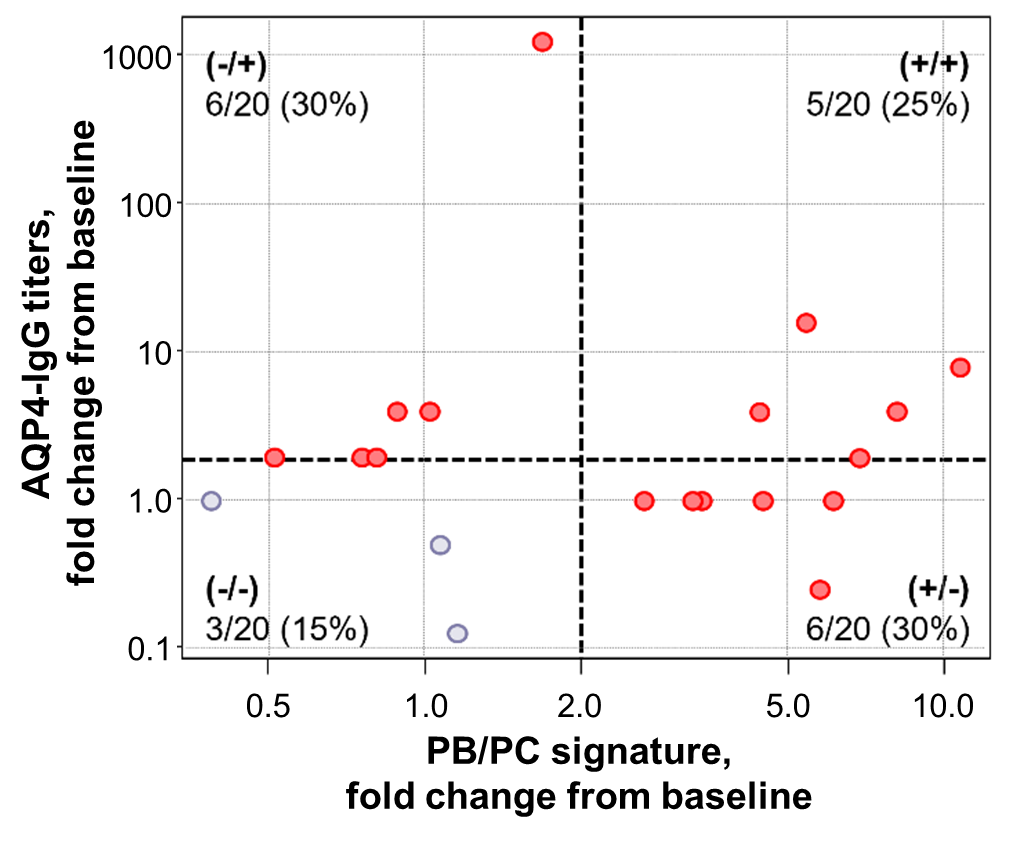


**Figure S6 A-B**. Increases in AQP4-IgG titers weakly correlate with increases in Transitional B cells and PC signature in participants receiving placebo during the N-MOmentum study.

**B**

**A**


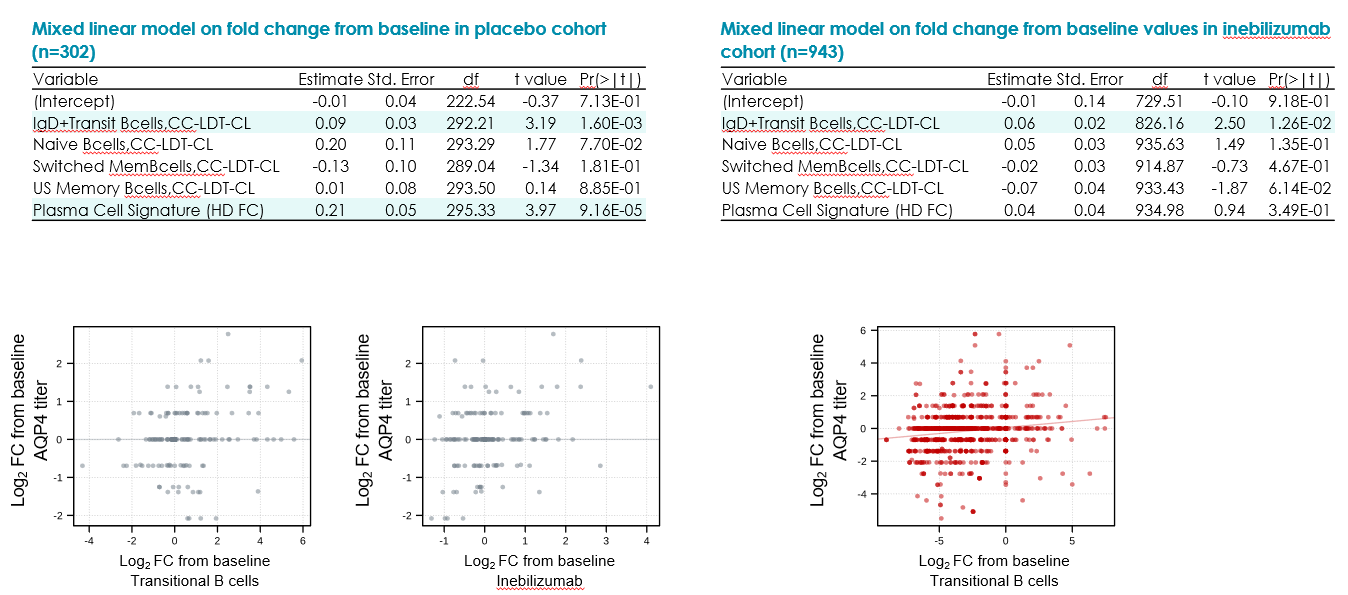


**Figure S7**. Immune profiles of eight participants who displayed the largest increases in the PC signature during the study. Presumably, if large increases in PC signature were observed, increases in AQP4-IgG would occur simultaneously or proceeding the increase in the PC signature. However, these increases in PC signature were largely not followed by increases in AQP4-IgG. This points to AQP4 Ig being largely secreted by CD19- long lived ASCs.


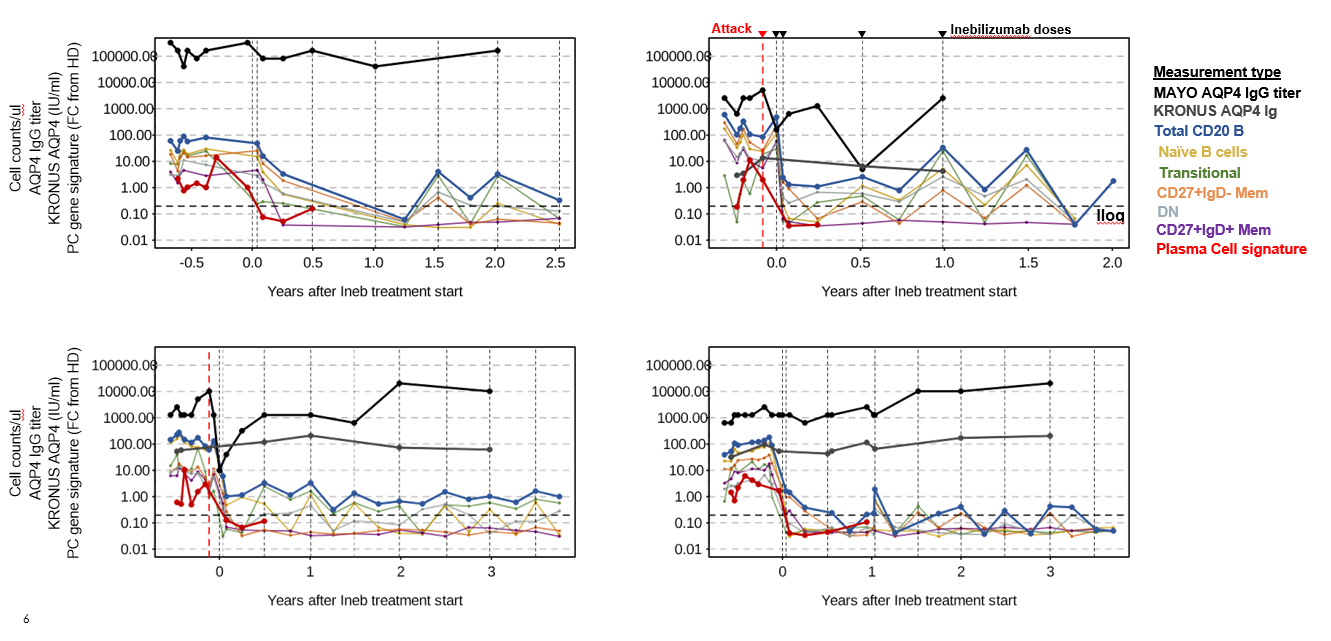


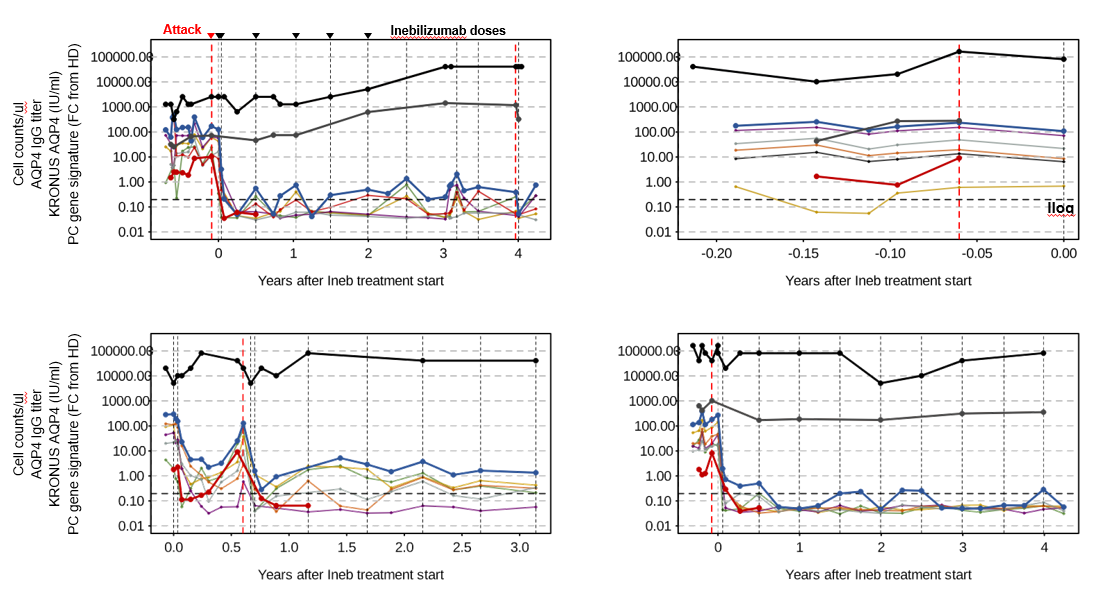

Supplement: Supplementary file 1 — Appendix S1. [file ACN3-11-2792-s001.docx]
